# Role of neopterin as an inflammatory biomarker in congestive heart failure with insights on effect of drug therapies on its level

**DOI:** 10.1007/s10787-022-01028-5

**Published:** 2022-07-25

**Authors:** Gaidaa M. Dogheim, Mohamed T. Amralla, Rehab H. Werida

**Affiliations:** 1 Pharmacy Practice Department, Faculty of Pharmacy, Alexandria University, Al Mesallah Sharq, Qism Bab Sharqi, Alexandria Governorate, Alexandria, 21500 Egypt; 2 Bachelor Degree, Faculty of Pharmacy, Alexandria University, Alexandria, 21500 Egypt; 3 Clinical Pharmacy and Pharmacy Practice, Faculty of Pharmacy, Damanhour University, Damanhour, 22514 Egypt

**Keywords:** Heart failure, Inflammation, Neopterin, Biomarker, Ivabradine

## Abstract

Inflammation has a major role in the pathogenesis of heart failure (HF). It triggers a cascade that leads to the release of pro-inflammatory cytokines which in turn cause cardiac hypertrophy, fibrosis, apoptosis, negative inotorpy and leukocyte recruitment which worsen the condition. Neopterin is an inflammatory biomarker which is released as a response to macrophage activation. Levels of neopterin are elevated in conditions which has an immunological component such as autoimmune disease, viral and bacterial infections and malignancy. Neopterin levels were found to be elevated in patients with HF. This is due to the fact that inflammation takes place during the development of the condition. Studies demonstrated that neopterin can be used as a biomarker for diagnosing HF, determining severity of the disease and monitoring its progression. Neopterin levels were higher in patients with New York Heart Association classification (NYHA) III–IV more than class I–II. Moreover, neopterin levels correlated well with morbidity and mortality. It has been suggested that neopterin be monitored levels to determine effectiveness of HF treatment options.

**Graphical abstract:**

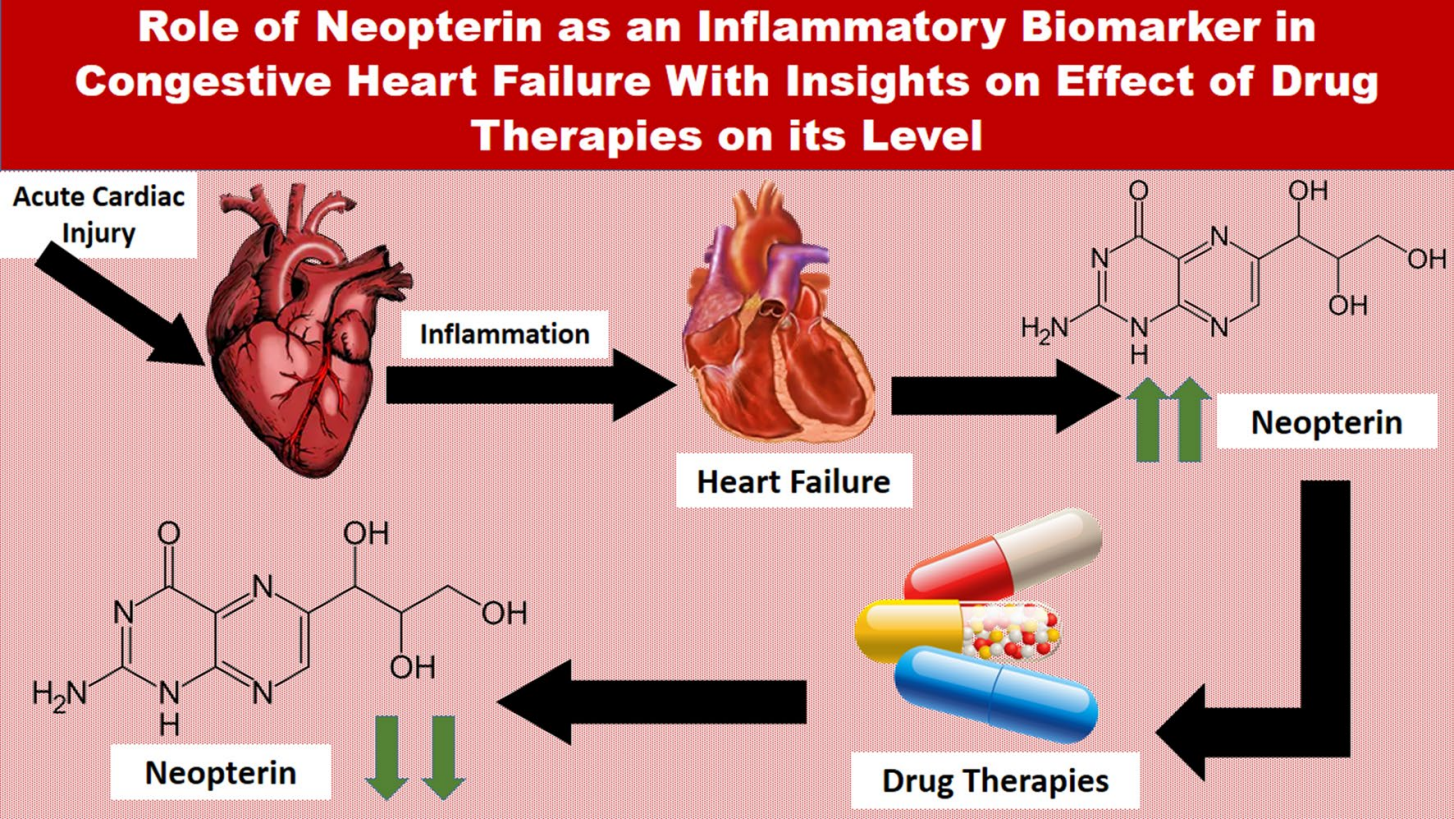

## Introduction

Heart failure (HF) is a chronic clinical condition where the heart fails to meet the demands of the body due to ventricular dysfunction (Morrissey et al. 2011; Ramani et al. 2010). The ventricles either fail to fill up properly resulting in heart failure with preserved ejection fraction (HFpEF) or fail to pump blood properly resulting in heart failure with reduced ejection fraction (HFrEF) (Morrissey et al. 2011; Ramani et al. 2010; Kemp and Conte 2012). Heart failure is classified according to New York Heart Association classification (NYHA) into stages from I to IV. The progressive nature of the disease results in poor patient quality of life and poor prognosis. Any abnormality affecting the cardiovascular system can result in heart failure. Hypertension, diabetes, ischemic heart disease, cardiomyopathy, valvular disease and arrhythmias are the most common causes of HF. (Ramani et al. 2010; Kemp and Conte 2012; Rich 1997).

Pathophysiology underlying heart failure can be explained through several models. It encompasses a combination of structural pathology, neurohormonal activation and end-organ dysfunction (Kemp and Conte 2012; Cohn 1996). In healthy subjects, the heart pumps blood to meet the body demands, which is known as the cardiac output (CO) and equivalent to 4-8L/min. Stroke volume is affected by three factors which are contractility, preload and afterload. Any changes in these factors will lead to decreased stroke volume and in return decreased cardiac output. The decrease in cardiac output triggers a cascade of events known as compensatory mechanism including sympathetic nervous system activation and renin–angiotensin–aldosterone system activation (RAAS) (Ramani et al. 2010; Kemp and Conte 2012). Sympathetic nervous system (SNS) releases noradrenaline (NA), leading to vasoconstriction and increased heart rate (HR) and contractility. Renin–angiotensin–aldosterone system releases angiotensin II, leading to vasoconstriction, and aldosterone, leading to salt and water retention (Ramani et al. 2010; Kemp and Conte 2012). By time, these compensatory mechanisms become burden on the cardiac myocytes, leading to further damage and worsening the condition. Neurohormonal activation also results in the release of certain substances such as natriuretic peptides, endothelin and neopterin (Kemp and Conte 2012). Endothelin is a strong vasodilator released from vascular endothelium. Natriuretic peptides such as brain natriuretic peptide (BNP), atrial natriuretic peptide (ANP) and C-type natriuretic peptide (CNP) lead to enhancing natriuresis and counteracting vasoconstricting effects of the SNS and RAAS (Kemp and Conte 2012).

A number of previous studies have demonstrated the role of inflammation in the pathophysiology of HF (Castillo et al. 2020; Adamo et al. 2020; Murphy et al. 2020; Dick and Epelman 2016). Moreover, inflammatory biomarkers such as tumor necrosis factor alpha (TNF-*α*), interferon gamma (INF-*γ*), interleukin 1-*β* (IL-1 *β*) and interleukin 6 (IL-6) have been used to predict the prognosis of HF (Shirazi et al. 2017; Libby et al. 2018). Neopterin—an inflammatory biomarker—is produced by activated macrophages in response to inflammation (Kaski et al. 2005; Pingle et al. 2008). Thus, in any condition involving immune response activation and inflammation, neopterin levels significantly increase. Neopterin levels have been found to be elevated in several diseases including certain malignancies, rheumatoid arthritis, viral infections, autoimmune diseases and coronary artery diseases (CAD) (Kaski et al. 2005; Pingle et al. 2008). Since the pathophysiology of heart failure includes an inflammatory response, levels of neopterin are supposed to be high in patients with heart failure. Yet, few studies investigated the possibility of such hypothesis. In this review, we discussed the role of neopterin as a biomarker in HF and effect of treatment on its level. Thus, the aim of this review is to discuss the role of neopterin as a biomarker in HF and demonstrate the relation between neopterin levels and HF drug therapies.

### Inflammation and pathogenesis of HF

Previous studies have demonstrated the role of inflammation in HF pathophysiology (Castillo et al. 2020; Adamo et al. 2020; Murphy et al. 2020; Dick and Epelman 2016). Any insult to cardiac myocytes leads to an inflammatory response to restore homeostasis and preserve cardiac function (Adamo et al. 2020). In case the inflammatory response persists, a state called “para-inflammation” takes place. Para-inflammation is a graded inflammatory response mainly present in the acute phase of cardiac injury (Adamo et al. 2020; Shirazi et al. 2017). It ranges from a physiological response to restore homeostasis and cardiac function to a sustained inflammation, leading to progressive left ventricular (LV) remodeling and dysfunction (Adamo et al. 2020). The pathogenesis of HF involves several inflammatory pathways. Cytokines levels have been found to be higher in HF patients. Pro-inflammatory cytokines and anti-inflammatory cytokines are two types of cytokines that function as opposing agents (Castillo et al. 2020; Dick and Epelman 2016; Shirazi et al. 2017). Pro-inflammatory cytokines lead to compensatory hypertrophy, fibrosis, apoptosis, negative inotorpy and leukocyte recruitment which contribute to further inflammation (Castillo et al. 2020; Dick and Epelman 2016; Shirazi et al. 2017). Examples of pro-inflammatory cytokines are TNF-*α*, INF-*γ*, IL-1*β*, IL-6, IL-17 and IL-18 (Castillo et al. 2020; Dick and Epelman 2016; Shirazi et al. 2017). Anti-inflammatory cytokines stimulate alternative macrophages pathway and proliferation of T-lymphocytes into T-helper 2 (Th-2) cells (Shirazi et al. 2017). This in turn leads to increased collagen synthesis, preserving cardiac function and inhibiting the action of pro-inflammatory cytokines (Shirazi et al. 2017). Anti-inflammatory cytokines include IL-4, IL-10 and transforming growth factor beta (TGF-β) (Castillo et al. 2020; Dick and Epelman 2016; Shirazi et al. 2017).

Since inflammation constitute a major role in the pathogenesis of HF, drug therapies targeted against inflammatory cytokines have been developed and tested to evaluate the option for their use in treatment of HF (Murphy et al. 2020). Options included anti-IL-1 therapy, methotrexate, colchicine and anti-IL-12/-23 therapy (Ustekinumab) (Murphy et al. 2020). Indirect anti-inflammatory therapies have been also used to improve the inflammation, thus improving the condition. Statins have known to have anti-inflammatory effects and can decrease levels of C-reactive protein (CRP) by 15–30% independent of lipid reduction (Jain and Ridker 2005). Moreover, statins counteract the effect of pro-inflammatory cytokines and enhance production of nitric oxide, leading to the improvement in HF condition (Jain and Ridker 2005). Guidelines-based anti-inflammatory therapeutic options include angiotensin converting enzymes (ACEIs) or angiotensin receptor blockers (ARBs), angiotensin receptor blockers/neprilysin inhibitor (ARNI) known as sacubitril/valsartan, mineralocorticoid antagonist (MRA) known as spironolactone and beta-blockers (McDonagh et al. 2021). These therapies counteract the inflammatory cascades that contribute to deterioration of HF, leading to improvement in the overall clinical condition.

### Neopterin as an inflammatory biomarker

Neopterin, 2-amino-4-hydroxy-6-(D-erythro-1ʹ, 2ʹ, 3ʹ-trihydroxypropyl)-pteridine, is biosynthetically derived from guanosine triphosphate (GTP) (Fuchs et al. 1992; Gieseg et al. 2018; Hamerlinck 1999; Murr et al. 2002). Neopterin is formed in macrophages after the induction of GTP cyclohydrolase I by INF-*γ* (Fuchs et al. 1992; Gieseg et al. 2018; Hamerlinck 1999; Murr et al. 2002). Thus, any condition that can induce production and activation of INF-*γ* can induce production of neopterin (Murr et al. 2002). There has been a correlation between increased INF-*γ* levels and high concentrations of neopterin (Murr et al. 2002). Since inflammatory response necessitate the production of INF-γ, neopterin levels are expected to be high in any inflammatory condition. Neopterin levels were high in several conditions such as viral and bacterial infections, autoimmune diseases, organ transplantation and malignancy (Fuchs et al. 1992; Murr et al. 2002). Recent studies evaluated the use of neopterin as a biomarker in monitoring the prognosis of various inflammatory disorders. Hence, neopterin offers a promising tool for monitoring the progression of various inflammatory conditions.

### Neopterin levels in cardiovascular diseases

Previous studies demonstrated the relation between neopterin levels and cardiovascular diseases. Zouridakis et al. demonstrated that high levels of neopterin were present in patients with stable angina and correlated with faster progression of the coronary artery disease (CAD) (Zouridakis et al. 2004). Avanzas et al. measured serum levels of neopterin in patients with chronic stable angina alongside other inflammatory markers (Avanzas et al. 2005). They demonstrated that levels of neopterin were high in chronic stable angina patients and correlated well with adverse coronary events (Avanzas et al. 2005). They stated that neopterin can be used as an independent predictor of coronary adverse events (Avanzas et al. 2005). Schumacher et al. examined levels of serum neopterin in patients with acute myocardial infarction (AMI) (Schumacher et al. 1997). They found that levels of neopterin was higher in patients with AMI than those with CAD or control subjects (Schumacher et al. 1997). Rodriguez et al. demonstrated that high levels of neopterin were associated with cardiac death and decreased ejection fraction (EF) in survivors of AMI (Dominguez-Rodriguez et al. 2006). Avanzas et al. measured levels of serum neopterin in hypertensive patients with chest pain (Avanzas et al. 2004). They stated that high levels of neopterin were found in patients with adverse events as progression of CAD as compared as those with no events (Avanzas et al. 2004). Erren et al. examined levels of neopterin in patients with coronary or peripheral atherosclerosis (Erren et al. 1999). They found that high levels of neopterin predict increased risk of CAD and/or stroke and transient ischemic attack (TIA) (Erren et al. 1999). Thus, neopterin can be used as a prognostic biomarker in cardiovascular disease.

### Neopterin in congestive heart failure

Many studies have proved strong correlation between neopterin levels and severity of heart failure. In 1993, a study was reported by Wiedermann et al. in which serum neopterin levels were measured in 16 heart failure patients compared to 11 healthy control individuals (Wiedermann et al. 1993). Investigators found out that serum levels of neopterin were high (≥ 12 nmol/L) in HF patients while controls had levels < 12 nmol/L (Wiedermann et al. 1993). Wietlicka-Kokoszanek et al. assessed neopterin as a biomarker for heart failure progression (Wietlicka-Kokoszanek et al. 2010). Serum neopterin levels were measured in 47, NYHA class II and III, hospitalized heart failure patients and 20 healthy control subjects (Wietlicka-Kokoszanek et al. 2010). The results showed a significant higher neopterin levels in the diseased group than the control group (Wietlicka-Kokoszanek et al. 2010). R.Caruso et al. looked into the relationship between neopterin concentrations in urine and left ventricular (LV) remodeling. Eight individuals with congestive heart failure (CHF) were compared to 19 healthy controls. The patients' group had considerably higher median neopterin levels (Caruso et al. 2013). In a cohort study by Shao et al., 53 ambulatory CHF patients underwent analysis for urine neopterin levels (Shao et al. 2014). Authors found a correlation between elevation of urine Neopterin levels and cardiac structural and functional abnormalities evaluated by echocardiography in those patients (Shao et al. 2014). Demir et al. evaluated in a prospective study the connection between serum neopterin levels and morbidity and mortality due to HF (Demir et al. 2019). Average neopterin serum concentration was significantly higher in the HF group compared to the control (Demir et al. 2019). Among HF patients, there was a significant statistical correlation between neopterin levels and rate of hospitalizations (Demir et al. 2019). Moreover, upon follow-up, 29 patients died where neopterin levels were higher as compared to those who survived (Demir et al. 2019). Furthermore, a study was reported by Yamamoto et al. comparing 68 hospitalized HF patients against healthy control individuals (Yamamoto et al. 2016). HF patients had significantly higher serum neopterin levels than controls (Yamamoto et al. 2016). Also, significant increase in neopterin serum concentration was observed when comparing patients with NYHA III/IV to patients with NYHA II (Yamamoto et al. 2016). The follow-up showed that high neopterin group among HF patients had greater chances to encounter cardiovascular events (including cardiovascular death, hospitalization due to HF or acute coronary syndrome) than the low-neopterin group (Yamamoto et al. 2016). Recently, it was proved by Lanser et al. that higher neopterin levels are associated with higher NYHA class in HF patients (Lanser et al. 2019). Moreover, they could prove that higher neopterin levels predict bad prognosis in HF patients such as death or hospitalization (Lanser et al. 2019). All these findings suggest that neopterin can be used as a biomarker of (CHF) severity and disease progression, indicating ongoing inflammatory activity in the deteriorating cardiac muscle.

### Neopterin versus BNP and NT-Pro BNP

Several studies demonstrated the applicability of BNP and NT-Pro BNP in diagnosis, prognosis and therapy guiding of HF (Bettencourt 2004; McDonagh et al. 2004; Seino et al. 2004; Murdoch et al. 1999). Bettencourt et al. established that NT-Pro BNP and BNP correlated well with the severity of HF, yet NT-Pro BNP had higher sensitivity than BNP (Bettencourt 2004). Moreover, both biomarker concentrations were high in patients with severe HF symptoms but only NT-Pro BNP predicted death or transplantation need (Bettencourt 2004). McDonough et al. demonstrated that NT-Pro BNP is a useful biomarker in the diagnosis of HF (McDonagh et al. 2004). Seino et al. proved that BNP and NT-Pro BNP levels reflect severity of HF yet NT-Pro BNP correlated better (Seino et al. 2004). Murdoch et al. demonstrated that ACEIs and diuretics use in HF patients lead to a decrease in both biomarkers levels which provide a useful tool for guiding therapy in HF (Murdoch et al. 1999). Yet, other studies showed that BNP and NT-Pro BNP are not specific nor selective markers for HF as their levels are elevated in other cardiac and non-cardiac conditions (Kim and Januzzi 2011; Silver et al. 2004; Ibrahim and Januzzi 2018). Silver et al. showed that levels of BNP are elevated in other cardiac conditions such as hypertrophic cardiomyopathy, diastolic dysfunction, left ventricular hypertrophy and dyspnea of respiratory disorder (Silver et al. 2004). Kim et al. demonstrated that biomarkers levels are elevated in acute coronary syndrome (ACS), myocarditis, cardioversion as well as other non-cardiac conditions (Kim and Januzzi 2011). Ibrahim et al. demonstrated that BNP and NT-Pro BNP levels are higher in chronic kidney disease than in HF independent from the presence of cardiovascular disease (Ibrahim and Januzzi 2018). On the other hand, studies showed that neopterin can be used as an independent biomarker for HF severity, diagnosis and prognosis (Caruso et al. 2013; Demir et al. 2019; Dogheim et al. 2022). Demir et al. demonstrated the diagnostic value of neopterin as they showed that there was no overlapping value between HF and control group (Demir et al. 2019). Moreover, they proved that neopterin correlated well with C-reactive protein (CRP) proving that it is a useful indicator of inflammation in HF (Demir et al. 2019). In addition, they proved that neopterin levels correlated with morbidity and mortality after 1-year follow-up as its level was higher in those with cardiovascular events than with those without (Demir et al. 2019). Caruso et al. proved that high neopterin levels correlated with severity of HF and degree of left ventricular remodeling, increase in cardiac volume and echocardiography values (Caruso et al. 2013). Moreover, they found a correlation between neopterin and IL-8 only reflecting the inflammatory component in HF (Caruso et al. 2013). They also demonstrated that CRP and NT-Pro BNP did not correlate with neopterin level (Caruso et al. 2013). Dogheim et al. showed that neopterin levels were high in patients with HF and levels were higher with NYHA class IV than III (Dogheim et al. 2022). They also demonstrated a correlation between neopterin with heart rate and NT-Pro BNP (Dogheim et al. 2022). All these data provide strong evidence about the reliability of using neopterin as a diagnostic and prognostic biomarker in HF.

### Heart failure treatment and neopterin levels

Significant progress has been achieved in HF treatment and various studies investigated the impact of drug therapies on levels of cardiac biomarkers. Unfortunately, only few reports investigated the impact of HF treatment on neopterin levels. Dogheim et al. investigated the effect of HF drug therapies on levels of neopterin (Dogheim et al. 2022). The researchers evaluated neopterin levels in two groups: one that received standard HF treatment (non-ivabradine group) and another that received ivabradine as a beta-blocker add-on or replacement medication (ivabradine group) (Dogheim et al. 2022). Levels of neopterin decreased significantly after 3 months of intervention with ivabradine (Dogheim et al. 2022). In non-ivabradine group, there was no significant change in neopterin levels (Dogheim et al. 2022). However, the study was only conducted for three months, and the sample size (*n* = 30) was rather small. Thus, further studies should be undertaken to assess the effect of HF treatment strategies on levels of neopterin.

In summary, neopterin levels are elevated in patients with HF and correlates well with disease severity. Moreover, neopterin can be used as a biomarker in the diagnosis and prognosis of HF. The use of neopterin to assess effectiveness of drug therapy in HF is yet to be further investigated to draw a definitive conclusion.

## Data Availability

There are no new data associated with this article.
